# The implications of autoantibodies to a single islet antigen in relatives with normal glucose tolerance: development of other autoantibodies and progression to type 1 diabetes

**DOI:** 10.1007/s00125-015-3830-2

**Published:** 2015-12-16

**Authors:** Polly J. Bingley, David C. Boulware, Jeffrey P. Krischer, the Type 1 Diabetes TrialNet Study Group

**Affiliations:** School of Clinical Sciences, University of Bristol, Learning and Research, Southmead Hospital, Bristol, BS10 5NB UK; Department of Pediatrics, Morsani College of Medicine, University of South Florida, Tampa, FL USA

**Keywords:** Autoantigens, Diabetes antibodies, GAD, HLA, IA-2, Insulin autoantibodies, Islet autoantibodies, Prediction, Prevention, Zinc transporter 8

## Abstract

**Aims/hypothesis:**

Autoantibodies directed at single islet autoantigens are associated with lower overall risk of type 1 diabetes than multiple autoantibodies, but individuals with one autoantibody may progress to higher risk categories. We examined the characteristics of this progression in relatives followed prospectively in the TrialNet Pathway to Prevention.

**Methods:**

The study population comprised 983 relatives who were single autoantibody positive with normal baseline glucose tolerance (median age 16.2 years). Samples were screened for antibodies to GAD, insulinoma-associated antigen 2 (IA-2) and insulin, and all positive samples tested for antibodies to zinc transporter 8 and islet cell antibodies.

**Results:**

Antibodies to at least one additional islet autoantigen appeared in 118 of 983 relatives (overall 5 year risk 22%, 95% CI [17.9, 26.1]). At baseline, antibodies to GAD alone (68%) were more frequent than antibodies to insulin (26%) or IA-2 (6%), but all were associated with a similar risk of developing additional autoantibodies. Risk was associated with younger age (*p* = 0.002) and HLA class II genotype, but was similar in high and intermediate genetic risk groups (*p* = 0.65). Relatives who became multiple autoantibody positive during the follow-up had increased risk of developing diabetes comparable with the risk in relatives with multiple autoantibodies at study entry.

**Conclusions/interpretation:**

Progression of islet autoimmunity in single autoantibody positive relatives in late childhood/adult life is associated with a predominance of autoantibodies to GAD and a distinct HLA risk profile. This heterogeneity in type 1 diabetes autoimmunity has potentially important implications for disease prevention.

**Electronic supplementary material:**

The online version of this article (doi:10.1007/s00125-015-3830-2) contains peer-reviewed but unedited supplementary material, which is available to authorised users.

## Introduction

The prodrome leading up to clinical onset of type 1 diabetes has been increasingly well characterised over the last three decades as the result of prospective study of relatives of people with the condition and particularly birth cohort studies in children with markers of high genetic risk [1]. We know that antibodies to islet autoantigens typically appear early in life, and may be present for some 30 years before diabetes develops. Individuals with antibodies to more than one islet autoantigen are at greatest risk and there is an increasing body of evidence that, in the long term, almost all people with multiple autoantibodies seem likely to develop diabetes [2–5]. In infants at high genetic risk, the progression from detection of a single islet autoantibody to the strongly disease-associated pattern of multiple autoantibody positivity and thence to overt diabetes usually occurs relatively rapidly [6–8]. In the combined dataset from BabyDiab, Diabetes Autoimmunity Study in the Young (DAISY) and Type 1 Diabetes Prediction and Prevention Project (DIPP) cohorts, the median age of seroconversion for multiple autoantibodies was 2.1 years, the cumulative risk of diabetes within 15 years of seroconversion was 84% and the median time to diabetes was 3.5 years [5]. We know that the risks of diabetes associated with a detection of antibodies to a single islet autoantigen are five- to eightfold lower than this and that some children develop single autoantibodies for the first time in later childhood and adolescence [2, 3, 6–9], but the natural history and determinants of progression to diabetes in this group are less well understood.

Our aim was to examine the characteristics of development of multiple islet autoantibodies and diabetes in single antibody positive relatives of people with type 1 diabetes taking part in a large prospective study. We set out to determine the risk of progression from single to multiple autoantibody positive status and to diabetes in a large cohort of relatives with normal glucose tolerance followed prospectively in TrialNet studies, and to examine the effect of demographic, genetic and autoantibody characteristics on these risks.

## Methods

Non-diabetic first, second and third degree relatives of people with type 1 diabetes were recruited to the TrialNet Natural History Study of the Development of Type 1 Diabetes (Pathway to Prevention [PTP]; ClinicalTrials.gov identifier: NCT00097292) as previously described [10]. All study participants gave informed consent and the study was approved by the responsible ethics committee for each study site. Participants were included in this analysis if they had antibodies to the same single islet autoantigen (GAD [GADA], insulin [IAA] or insulinoma-associated antigen 2/ICA512 [IA-2A]) on at least two occasions and antibody results were available from at least one subsequent study visit. All samples were screened for GADA, IAA and IA-2A and, if levels any of these were above the threshold of positivity, testing for islet cell antibodies (ICA) and antibodies to zinc transporter 8 (ZnT8A) was added. Individuals with confirmed islet autoantibodies underwent baseline assessment including oral glucose tolerance testing and were followed 6–12 monthly in accordance with the PTP study protocol. From 2004 to 2012, single autoantibody positive relatives were invited for 6-monthly visits including antibody determination and OGTT. Since 2012, single autoantibody positive relatives with normal glucose tolerance, HbA_1c_ and a diabetes low risk score [11] have been followed with 12-monthly islet autoantibody testing and HbA_1c_ determination. Individuals with abnormal glucose tolerance at baseline and those carrying the protective HLA class II haplotype DQA1*01:02-DQB1*06:02 were excluded from the analysis.

### Assays

GADA, IAA, IA-2A and ZnT8A were measured by radioimmunoassay in the TrialNet Core laboratory at the Barbara Davis Center for Childhood Diabetes (BDC) as previously described [9]. Up to 2010, antibodies to GAD and ICA512 were tested in a combined assay using ^3^H-leucine-labelled GAD_65_ and ^35^S-methionine-labelled ICA512—the ‘BDC in-house’ assay—with results expressed as an index. Since June 2010, the laboratory has used the harmonised GADA and IA-2A assays for National Institute of Diabetes and Digestive and Kidney Diseases (NIDDK) Consortia [12]. The major differences are that, in the harmonised assays: (1) the antibodies are measured separately using ^35^S-methionine-labelled in vitro transcribed and translated GAD_65_ and IA-2; (2) results are expressed in Digestive and Kidney (DK) units/ml derived from standard curves made up of dilutions of common positive and negative NIDDK working calibrators; and (3) thresholds have been defined as equivalent to the 97th percentile in 500 adult blood donor controls. In a comparison of the BDC in-house and harmonised assays in 2,170 TrialNet PTP samples, designation of positive/negative status was 96% concordant for GADA and 95% concordant for antibodies to ICA512/IA-2A. GAD and IA-2/ICA512 antibody positive status was based on the results of harmonised assays if available, and otherwise on the in-house BDC assay. ICA were assayed by indirect immunofluorescence at the University of Florida (Gainesville, FL, USA). Assay quality assurance is under regular review by the TrialNet Laboratory Monitoring Committee. In the 2012 Islet Autoantibody Standardization Programme proficiency evaluation, the BDC in-house assays for GADA, ICA512/IA-2A, IAA and ZnT8A achieved 64%, 60%, 50% and 62% sensitivity with 100%, 100%, 100% and 98% specificity, respectively. The harmonised GADA and IA-2A assays achieved 66% and 70% sensitivity with 99% and 100% specificity, respectively.

HLA-DQ polymorphisms were determined by allele-specific oligonucleotide genotyping [9, 13]. The haplotypes of interest were DQA1*05:01-DQB1*02:01 (*DQ2*), DQA1*03:01-DQB1*03:02 (*DQ8*) and DQA1*01:02-DQB1*06:02 (*DQ6*). HLA class II genotypes were defined as high (*DQ2/DQ8*), moderate (*DQ2/DQ2*, *DQ8/DQ8* or *DQ8/X*) or low risk (*DQ6/X*, *DQ2/X* or *DQX/X*) where X represents a haplotype other than *DQ2* or *DQ8*.

### Statistical analysis

The main outcome was the confirmed development of multiple autoantibodies defined as detection on two occasions of at least two of the five islet autoantibodies included in the testing strategy (GADA, IAA, IA-2A, ZnT8A and ICA). Diabetes was defined by WHO criteria. Time-to-event analysis using the Kaplan–Meier method was done to examine progression from single autoantibody positivity to development of multiple autoantibodies and diabetes, as well as from development of multiple antibodies to diabetes. The time-to-event was calculated from the date of first detection of a single islet autoantibody to date of first detection of multiple autoantibodies, diagnosis of diabetes or last follow-up. Logrank testing was used to compare the cumulative incidence of development of multiple autoantibodies or diabetes between groups. Time to onset of development of multiple autoantibodies by individual and combined risk markers, including age at initial screening, sex, race, ethnicity, relationship to the proband, antibody type and HLA class II genotype was assessed by Cox proportional hazards regression model. The effect of initial antibody titre was further analysed within subgroups defined by antibody type. Stratified Kaplan–Meier curves were generated for variables shown to be significant in the multivariable model. For continuous variables (age and antibody titre), optimal cutpoints were identified using forward stepwise Cox proportional hazard regression models over a range of possible thresholds. For each variable the optimal cutpoint was that giving the highest level of significance. Analyses including GADA and IA-2A titre were restricted to samples for which BDC in-house assay results were available.

## Results

Of 119,074 relatives screened in the TrialNet PTP by April 2013, 1,195 had confirmed antibodies to only one islet autoantigen (GADA, IAA or IA-2A/ICA512 without ICA or ZnT8A). Of these, 983 had normal glucose tolerance at baseline and were therefore eligible for inclusion in the analysis. The median age of these participants was 16.2 years (interquartile range 8.7–36.2), 60% were female and 85% were white. Of the 983 relatives, 672 (68%) were positive for GADA, 252 (26%) for IAA and 59 (6%) for IA-2A. Other characteristics are given in Table 1.

**Table 1:** Participant characteristics

	Single, confirmed antibody positive (983 individuals)	Progressed to confirmed multiple antibody positive (118 individuals)
Age at screening		
Median (interquartile range)	16.2 (8.7–36.2)	11.0 (6.5–24.0)
<6 years	138 (14.0)	28 (23.7)
6–12 years	279 (28.4)	47 (39.8)
13–24 years	167 (17.0)	14 (11.9)
≥25 years	399 (40.6)	29 (24.6)
Sex		
Male	394 (40.3)	60 (50.8)
Female	583 (59.7)	58 (49.2)
Race		
White	812 (84.8)	111 (96.5)
Non-white	146 (15.2)	4 (3.5)
Ethnicity		
Hispanic or Latino	151 (15.6)	4 (3.5)
Not Hispanic or Latino	793 (82.1)	110 (95.6)
Unknown	22 (2.3)	1 (0.9)
Relation to the type 1 diabetic proband		
Sibling	390 (40.1)	65 (56.0)
Offspring	166 (17.1)	24 (20.7)
Other^a^	114 (11.7)	8 (6.9)
Parent	302 (31.1)	19 (16.4)
HLA risk		
High: *DQ2/DQ8*	110 (13.6)	20 (18.2)
Moderate: *DQ2/DQ2*, *DQ8/DQ8* or *DQ8/DQX*	311 (38.5)	55 (50.0)
Low: *DQX/DQX* or *DQ2/DQX* ^b^	387 (47.9)	35 (31.8)

All values are *n* (%) unless otherwise indicated

^a^Second or third degree relatives of a person with type 1 diabetes and, aged ≤20 years at study entry

^b^Individuals with *DQ6* genotypes were excluded from the analysis

### Development of additional autoantibodies

**Fig. 1:**
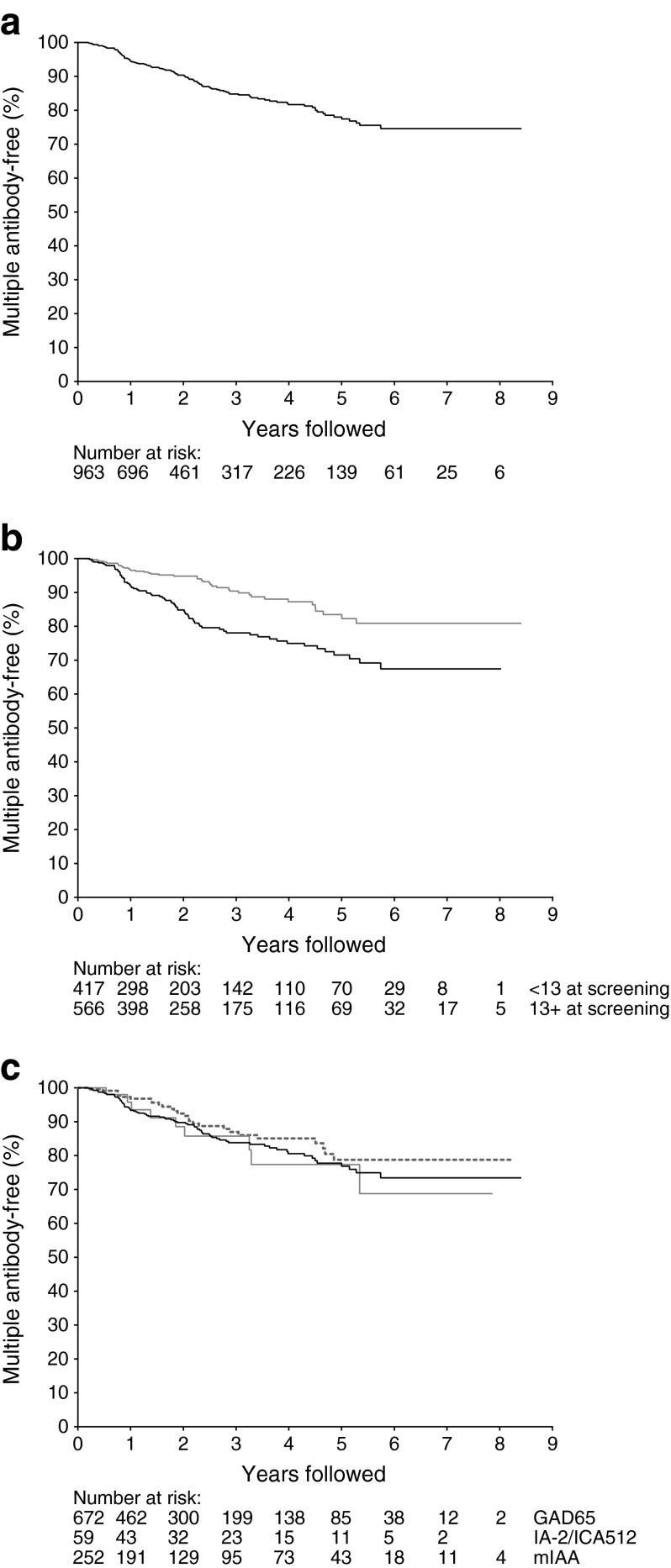
(**a**) Time from initial detection of confirmed antibodies to a single islet antigen (GAD, IA-2/ICA512 or insulin) to first detection of confirmed multiple antibodies. The number of individuals at risk at the start of each year of follow-up is shown below each graph. (**b**) Time from initial detection of confirmed antibodies to a single islet antigen (GAD, IA-2/ICA512 or insulin) to first detection of confirmed multiple antibodies in relatives aged <13 years (black line) and ≥13 years (grey line) (logrank test, *p* < 0.001). (**c**) Time from initial detection of confirmed antibodies to a single islet antigen (GAD [black line], IA-2/ICA512 [grey line] or insulin [dashed line]) to first detection of confirmed multiple antibodies (logrank test, *p* = 0.38)

The median follow-up of the cohort was 2.2 years, during which 118 relatives with confirmed single autoantibody positivity developed antibodies to at least one additional islet autoantigen. Of these, 82 were GADA positive in the initial sample, 27 were IAA positive and nine IA-2A positive. The time from initial confirmed single islet autoantibody to first confirmed detection of at least one additional autoantibody is shown in Fig. 1a, and details of the additional autoantibodies detected are given in electronic supplementary material (ESM) Table 1. Among the 118 relatives who developed additional autoantibodies, 44 were categorised as multiple autoantibody positive only on the basis of detection of ICA in follow-up samples; 40 with GADA and ICA, and four with IAA and ICA. The median age at detection of the second autoantibody was 12.7 (7.8–26.4). The overall 5 year risk of becoming positive for multiple autoantibodies was 22.0% (95% CI [17.9, 26.1]). Risks were higher in younger participants (Fig. 1b). The 5 year-risk below the optimal cutpoint for age, 13 years, was 28.5% (22.2, 34.8) compared with 16.5% (11.0, 22.0) in relatives above age 13 (*p* < 0.001); 25.9% (19.4, 32.4) in males and 19.5% (14.2, 24.8) in females (*p* = 0.008); 23.9% (19.4, 28.4) in white individuals compared with 10.1% (0, 21.5) in non-white individuals (*p* = 0.002); and 37.6% (20.4, 54.8), 30.8% (22.8, 38.8) and 17.0% (11.3, 22.7), respectively, in individuals with high, moderate and low risk HLA class II genotypes (*p* = 0.001). Initial antibody type did not influence the risk of developing additional autoantibodies; risks were similar in the groups with antibodies to GAD, insulin or IA-2/ICA512 (Fig. 1c). The results of Cox proportional hazards regression are shown in Table 2. On multivariable analysis, age at screening, race and HLA class II genotype, but not sex, were confirmed to be independent determinants of risk.

**Table 2:** Cox proportional hazards regression for time from initial confirmed single autoantibody positive to first confirmed multiple autoantibody positive (overall *n* = 983)

	Univariate HR (95% CI)	*p* value	Multivariable HR (95% CI)	*p* value
Age at screening	0.97 (0.96, 0.99)	<0.001	0.96 (0.93, 0.98)	0.002
Sex				
Male	1.63 (1.13, 2.33)	0.008	1.28 (0.86, 1.90)	0.23
Female	Ref.		Ref.	
Race				
White	4.37 (1.61, 11.84)	0.004	4.23 (1.23, 14.53)	0.02
Non-white	Ref.		Ref.	
Ethnicity				
Hispanic or Latino	0.20 (0.08, 0.55)	0.002	0.31 (0.10, 1.02)	0.05
Not Hispanic or Latino	Ref.		Ref.	
Unknown	0.45 (0.06, 3.24)	0.43	2.06 (0.25, 17.24)	0.50
Relation to type 1 diabetic proband				
Sibling	2.83 (1.70, 4.72)	<0.0001	1.03 (0.40, 2.52)	0.99
Offspring	2.12 (1.16, 3.87)	0.01	0.80 (0.30, 2.16)	0.66
Other^a^	0.93 (0.41, 2.13)	0.87	0.48 (0.15, 1.56)	0.22
Parent	Ref.		Ref.	
Single antibody type				
GADA	Ref.		Ref.	
IA-2A/ICA512	1.09 (0.54, 2.16)	0.81	1.29 (0.62, 2.70)	0.50
IAA	0.75 (0.48, 1.15)	0.19	0.70 (0.44, 1.12)	0.14
HLA risk				
High: *DQ2/DQ8*	2.24 (1.29, 3.88)	0.004	1.98 (1.11, 3.52)	
Moderate: *DQ2/DQ2*, *DQ8/DQ8* or *DQ8/DQX*	2.00 (1.31, 3.06)	0.001	1.89 (1.21, 2.94)	0.02
Low: *DQX/X* or *DQ2/X* ^b^	Ref.		Ref.	0.005

^a^Second or third degree relatives of a person with type 1 diabetes, aged ≤20 years at study entry

^b^Individuals with *DQ6* genotypes were excluded from the analysis

Ref., reference

In 450 GADA positive individuals for whom BDC in-house assay results were available, risk of developing additional autoantibodies was influenced by initial antibody titre (multivariable HR 4.4 [2.0, 9.5], *p* < 0.001). The 5 year risk in those with GADA index ≥0.2 (the optimal standard assay cutpoint) was 45.3% (34.9, 55.7) compared with 8.4% (4.5, 12.3) in those with GADA index <0.2 (*p* < 0.001). A similar effect was seen in 229 individuals with harmonised GADA assay results though the median duration of follow-up was shorter (0.88 years); the risk of developing additional autoantibodies within 2 years was 33.1% (1.2, 65.0) in participants with GADA ≥220 DK units/ml (the optimal harmonised assay cutpoint) compared with 4.9% (0.6, 9.2) with GADA <220 DK units/ml (*p* = 0.001). Associations between IAA and IA-2A titres and risk of becoming multiple autoantibody positive could not be assessed owing to the small number of individuals who developed additional autoantibodies.

Risk of developing additional antibodies was similar in participants carrying high compared with moderate risk HLA class II genotypes (multivariable HR 1.13 [0.68, 1.88], *p* = 0.65). Among those with moderate risk genotypes, the 5 year risk did not differ between those carrying *DQ2/DQ2* and *DQ8/DQ8* (33% [11, 54] and 56% [36, 77], respectively, *p* = 0.16).

### Progression to diabetes

**Fig. 2:**
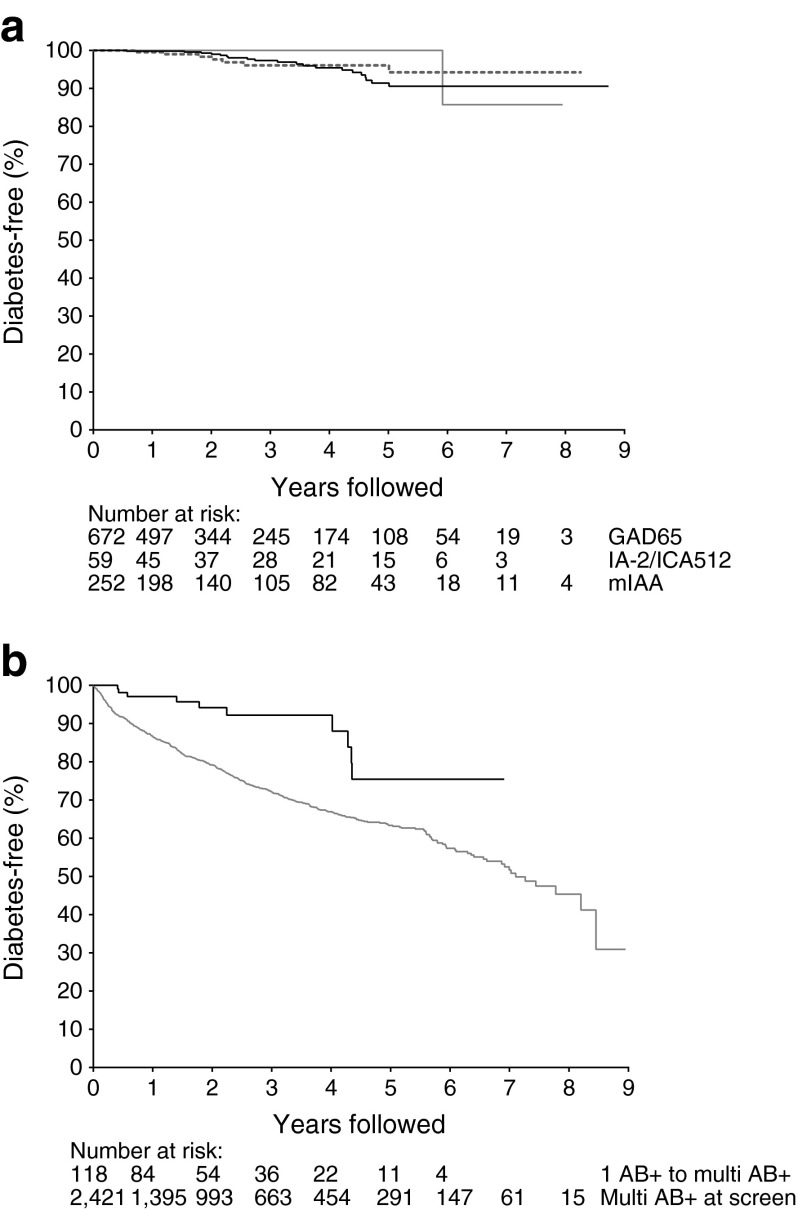
(**a**) Progression to type 1 diabetes in relatives positive for antibodies to GAD (black line), insulin (dashed line) and IA-2/ICA512 (grey line) (logrank test, *p* = 0.662). (**b**) Progression to type 1 diabetes in relatives who were confirmed multiple antibody positive at screening (grey line) and those who progressed from confirmed single antibody positive to confirmed multiple antibody positive from the time of progression (black line) (logrank test, *p* < 0.001)

A total of 28 single autoantibody positive relatives with normal glucose tolerance at baseline progressed to diabetes. Of these, 20 (71%) were GADA positive in the initial sample, 7 (25%) were IAA positive and 1 (4%) was IA-2A positive. The median time to diagnosis was 2.7 years. The overall 5 year risk of developing diabetes was 6.6% (3.9, 9.3); and did not differ between participants with GADA (6% [4.7, 12.5]), IAA (3.9% [0.8, 7.0]) or IA-2A (0%) at baseline (*p* = 0.66) (Fig. 2a). Of those who developed diabetes, ten were multiple autoantibody positive in at least one follow-up sample prior to diagnosis. The 5 year risk of diabetes after first detection of multiple islet autoantibodies in previously single antibody positive relatives was 24.5% (8.8, 40.2), compared with 5.7% (3.0, 8.4) in participants who remained positive for only one autoantibody (*p* < 0.0001), and was not different from the 5 year risk in participants in the PTP study who were found to have multiple antibodies at the initial screening visit (36.8% ([33.9, 39.7], *p* = 0.06)) (Fig. 2b).

## Discussion

In this study, we found that relatives persistently positive for antibodies to a single islet autoantigen had a 22% cumulative risk of progressing to the more strongly disease-associated pattern with antibodies to multiple autoantigens within 5 years. Development of additional autoantibodies was not restricted to early childhood, but occurred also in late childhood and adult life. Risk was not influenced by initial autoantibody type, but was related to antibody titre among those with GADA. While the overall risk of early progression to diabetes among single autoantibody positive relatives was relatively low, we have shown that it was higher in those who developed additional antibodies.

A major strength of this study is the size of the study cohort followed up 6–12 monthly using a standard protocol. The wide age range of the participants and the inclusion of relatives carrying low and intermediate as well as high risk HLA class genotypes mean that our findings complement those from prospective studies from birth. Our study does, however, have some limitations. The TrialNet PTP study protocol meant that rescreening was not offered to adults if no antibodies were detected in the initial samples and, even in children, uptake for rescreening was relatively low. Some relatives who were initially autoantibody negative relatives but subsequently seroconverted to autoantibody positivity might therefore have been missed. In addition, because the cohort was not followed from birth, we are not able to exclude the possibility that some of the participants were previously positive for other autoantibodies as well as those detected in the first and confirmatory study samples. A further consideration is that the median duration of follow-up of the cohort is still relatively short. All antibody testing was done in core reference laboratories with robust quality assurance of long-term assay performance. The change in GAD and IA-2/ICA512 antibody assays during the course of the study does represent a potential weakness, but was addressed by undertaking a large validation exercise which demonstrated good concordance between the original and new harmonised assays. Furthermore, the limited analysis possible in subgroups of relatives identified as single antibody positive using original and harmonised assays, showed similar risk of developing additional antibodies (data not shown). Because the harmonised GADA assay is generally more sensitive than the BDC in-house ^3^H-leucine-labelled GAD_65_ assay used in the early years of the study, the ‘appearance’ of GADA for the first time in follow-up samples could potentially be an artefact of the change of assay. We are, however, able to quantify the possible impact of this and show that it would affect a maximum of 12 of the 118 participants whom we have defined as progressing to multiple autoantibody positivity (data not shown). The optimal cutpoints for age and antibody titre that we have used are empirical and would need to be validated in an independent dataset if they were to be used for selection of subgroups in future studies. Finally, as an international consortium, TrialNet is not population-based and our study cohort was heterogeneous but, although the findings may not be applicable in all populations, we believe that the relatives recruited to the study are likely to be representative of the potential participants in future prevention studies.

Birth cohort studies have greatly clarified the natural history of islet immunity in early life, demonstrating that, in populations at high genetic risk, autoantibodies generally appear in infancy with rapid evolution of the broad immunoreactivity typical of the disease-associated humoral immune response characterised by antibodies to multiple islet autoantigens [6–8]. The implications of antibodies to a single autoantigen that appear in infancy but do not progress to the broader response or are detected for the first time in childhood are however less clear. Previous prospective studies have shown that risk of progression to diabetes was substantially lower in relatives who were positive for a single islet antibody than in those with two or more antibodies. The earliest reports relied on initial screening for ICA by indirect immunofluorescence and found that relatives with ICA alone were at similar risk to those who were antibody negative [2, 14]. Comparable results were subsequently seen for isolated antibodies to GAD, IA-2/ICA512 or insulin in a number of family study populations [15–17], when samples from relatives screened for Diabetes Prevention Trial type 1 (DPT-1) were re-tested for these [3] and in 1,533 single antibody positive relatives included in an earlier TrialNet analysis [9]. It is, however, clear that some individuals found to have single antibodies at initial screening, and some previously antibody negative relatives who develop antibodies for the first time in later childhood or adult life [18], do progress to diabetes. This analysis is, to our knowledge, the first to characterise these in detail.

We have shown that progression from one stage of autoimmunity to the next is not restricted to early childhood, but that there appears to be a complementary, less aggressive pathway for development of islet autoimmunity and risk of type 1 diabetes that continues beyond the first few years of life. In contrast to the ‘classic’ pathway seen in infancy, in which the first autoantibodies are generally directed against insulin, this alternative route appears to start with autoimmunity directed against GAD. Progression is associated with *HLA-DR3-DQ2* and -*DR4-DQ8* haplotypes, but while conversion to multiple antibodies early in life has been found to be strongly associated with *DQ2/DQ8* and *DQ8/DQ8* genotypes [6], the risk in this older cohort is similar between high and moderate risk HLA genotypes analysed. This concept of heterogeneity in the autoimmune process is consistent with recent evidence from The Environmental Determinants of Diabetes in the Young study that, in infancy, the initiation of autoimmunity to GAD and insulin differ in both timing and HLA associations [19]. In addition, two distinct immunological phenotypes—proinflammatory and partially regulated—have been identified using multiparameter analysis of autoantibody and autoreactive T cell responses, complemented by examination of the immunohistological characteristics of insulitis in pancreas tissue collected from children and adolescents who died soon after diagnosis of type 1 diabetes [20].

Clearer definition of heterogeneity in the process leading to type 1 diabetes and identifying the underlying mechanisms have potentially important implications for disease prevention, offering the possibility to target immunointervention strategies more precisely [21]. It is estimated that some 50% of type 1 diabetes is diagnosed after age 20 years [22] and it is possible that the slower course of adult onset type 1 diabetes results from this alternative pathway.

A further implication is that this stepwise process offers the opportunity to select intermediate endpoints for use in the design of type 1 diabetes prevention trials [23] and therefore to maximise the yield from screening relatives by offering a variety of studies suitable for groups at different levels of risk. We have demonstrated that transition from single to multiple antibodies represents a progression in the disease process that is associated with increased risk of clinical onset of diabetes. Based on the results of this analysis, a placebo controlled study that recruited around 280 relatives aged 8–20 years who are positive for GADA alone would have 80% power to detect a 50% reduction in the observed rate of progression to multiple antibodies over 5 years. Other autoantibody characteristics, such as affinity and epitope specificity, may allow further stratification of risk and facilitate the design and implementation of prevention trials [24, 25]. The recently described electrochemiluminescence assays for IAA and GADA and substituting N-terminally truncated GAD_65_ (95–585) for full-length GAD in the harmonised radiobinding assay have been shown to improve disease specificity and more accurately discriminate risk of progression to diabetes in single autoantibody positive individuals [26–28]. More detailed comparison of the genetic characteristics—both HLA and non-MHC—between the single autoantibody positive individuals who develop additional autoantibodies and those who do not may also refine risk assessment and provide insights into the underlying autoimmune process.

In summary, we have demonstrated that a subset of relatives positive for a single islet autoantibody is at risk of progression of autoimmunity with the appearance of additional autoantibodies in late childhood and adult life, and that this change is associated with increased risk of diabetes. This offers the opportunity to design innovative trials that complement those intervening in established advanced autoimmunity or aiming to prevent its initiation.

## Electronic supplementary material

ESM Table 1 (PDF 10 kb)

ESM Appendix (PDF 172 kb)

## Abbreviations

BDC: Barbara Davis Center for Childhood Diabetes
DK: Digestive and Kidney
GADA: Antibodies to GAD
IA-2: Insulinoma-associated antigen 2
IA-2A: Antibodies to insulinoma-associated antigen 2
IAA: Insulin autoantibodies
ICA: Islet cell antibodies
NIDDK: National Institute of Diabetes and Digestive and Kidney Diseases
PTP: Pathway to Prevention
ZnT8A: Antibodies to zinc transporter 8
