# Supplementary material for: The implications of autoantibodies to a single islet antigen in relatives with normal glucose tolerance: development of other autoantibodies and progression to type 1 diabetes
Source: Diabetologia. 2015 Dec 16;59:542–9. doi: 10.1007/s00125-015-3830-2 (PMC4742489; doi:10.1007/s00125-015-3830-2)
Supplement: Supplementary file 1 — (PDF 10 kb) [file 125_2015_3830_MOESM1_ESM.pdf]

ESM Supplementary Table: Islet autoantibodies detected in follow-up samples in 118 single antibody positive individuals who developed confirmed multiple autoantibodies

Islet autoantibodies detected in follow-up samples	*n*	*First islet autoantibodies detected*		
		GADA	IAA	IA-2A/ICA512
GADA/IAA	20	8	12	
GADA/IA-2A	8	5	1	2
GADA/ZnT8A	7	7		
GADA/ICA	40	40		
IAA/IA-2A				
IAA/ZnT8A				
IAA/ICA	4	1	3	
IA-2A/ZnT8A	1			1
IA-2A/ICA	2			2
GADA/IAA/IA-2A	2		2	
GADA/IAA/ZnT8A				
GADA/IAA/ICA	5	2	3	
GADA/IA-2A/ZnT8A	2	2		
GADA/IA-2A/ICA	6	4		2
GADA/ZnT8A/ICA	2	2		
IAA/IA-2A/ZnT8A				
IAA/IA-2A/ICA				
IA-2A/ZnT8A/ICA	1			1
GADA/IAA/IA-2A/ZnT8A				
GADA/IAA/IA-2A/ICA	4	2	2	
GADA/IAA/ZnT8A/ICA	3	1	2	
GADA/IA-2A/ZnT8A/ICA	8	7		1
IAA/IA-2A/ZnT8A/ICA	1		1	
GADA/IAA/IA-2A/ZnT8A/ICA	2	1	1	
	118	82	27	9
