# Supplementary material for: The implications of autoantibodies to a single islet antigen in relatives with normal glucose tolerance: development of other autoantibodies and progression to type 1 diabetes
Source: Diabetologia. 2015 Dec 16;59:542–9. doi: 10.1007/s00125-015-3830-2 (PMC4742489; doi:10.1007/s00125-015-3830-2)
Supplement: Supplementary file 2 — (PDF 172 kb) [file 125_2015_3830_MOESM2_ESM.pdf]

***ESM Appendix:* The Type 1 Diabetes TrialNet Study Group**
*(personnel as of 30th April 2015)*

Steering Committee: J.S. Skyler (University of Miami, Chair), M. Anderson (University of California, San Francisco), P. Antinozzi (Wake Forest University), M. Atkinson (University of Florida), M. Battaglia (San Raffaele University), D. Becker (University of Pittsburgh), P. Bingley (University of Bristol), E. Bosi (San Raffaele University), J. Buckner (Benaroya Research Institute), P. Colman (Walter & Eliza Hall Institute of Medical Research), L. DiMeglio (Indiana University), S. Gitelman, (University of California, San Francisco), R. Goland (Columbia University), P. Gottlieb (Barbara Davis Center for Childhood Diabetes), C. Greenbaum (Benaroya Research Institute), K. Herold (Yale University), R. Insel (Juvenile Diabetes Research Foundation), T. Kay (St Vincent's Institute of Medical Research), M. Knip (University of Helsinki), J. Krischer (University of South Florida), A. Lernmark (Skane University), J.B. Marks (University of Miami), A. Moran (University of Minnesota), J. Palmer (University of Washington), M. Peakman (King's College), L. Philipson (University of Chicago), A. Pugliese (University of Miami), P. Raskin (University of Texas Southwestern), M. Redondo (Baylor University), H. Rodriguez (University of South Florida), B. Roep (Leiden University Medical Center), W. Russell (Vanderbilt University), L. Spain (National Institute of Diabetes and Digestive and Kidney Diseases [NIDDK]), D.A. Schatz (University of Florida), J. Sosenko (University of Miami), D. Wherrett (University of Toronto), D. Wilson (Stanford University), W. Winter (University of Florida), A. Ziegler (Forschergruppe Diabetes); Previous Members: C. Benoist (Joslin Diabetes Center), J. Blum (Indiana University), K. Bourcier, P. Chase (Barbara Davis Center for Childhood Diabetes), M. Clare Salzler (University of Florida), R. Clynes (Columbia University), G. Eisenbarth (Barbara Davis Center for Childhood Diabetes), C. G. Fathman (Stanford University), G. Grave (National Institute of Child Health and Human Development), B. Hering (University of Minnesota), F. Kaufman (Children's Hospital Los Angeles), E. Leschek (NIDDK), J. Mahon (University of Western Ontario), K. Nanto Salonen (University of Turku), G. Nepom (Benaroya Research Institute), T. Orban (Joslin Diabetes Center), R. Parkman (Children's Hospital Los Angeles), M. Pescovitz (Indiana University), J. Peyman (National Institute of Allergy and Infectious Disease), M. Roncarolo (San Raffaele University), P. Savage (NIDDK), O. Simell (University of Turku), R. Sherwin (Yale University), M. Siegelman (University of Texas Southwestern), A. Steck (Barbara Davis Center for Childhood Diabetes), J. Thomas (Vanderbilt University), M. Trucco (University of Pittsburgh), J. Wagner (University of Minnesota).

Executive Committee: Jay S. Skyler, Katarzyna Bourcier, Carla J. Greenbaum, Richard Insel, Jeffrey P. Krischer, Ellen Leschek, Lisa Rafkin, Lisa Spain. Past Members: Catherine Cowie, Mary Foulkes, Heidi Krause-Steinrauf, John M. Lachin, Saul Malozowski, John Peyman, John Ridge, Peter Savage, Stephanie J. Zafonte.

Chairman's Office: Jay S. Skyler, Carla J. Greenbaum , Norma S. Kenyon, Lisa Rafkin, Irene Santiago, Jay M. Sosenko.

TrialNet Coordinating Center (University of South Florida): Jeffrey P. Krischer, Brian Bundy, Michael Abbondondolo, Timothy Adams, Darlene Amado, Ilma Asif, Matthew Boonstra, David Boulware, Brian Bundy, Cristina Burroughs, David Cuthbertson, Mary Deemer, Christopher Eberhard, Steve Fiske, Julie Ford, Jennifer Garmeson, Heather Guillette, Susan Geyer, Brian Hays, Courtney Henderson, Martha Henry, Kathleen Heyman, Belinda Hsiao, Christina Karges, Nichole Keaton, Amanda Kinderman, Pat Law, Ashely Leinbach, Cristin Linton, Shu Liu, Jennifer Lloyd, Jamie Malloy, Kristin Maddox, Julie Martin, Jessica Miller, Eric Milliot, Margaret Moore, Sarah Muller, Thuy Nguyen, Ryan O'Donnell, Vanessa Oduah, Jennifer Pilger, Amy Roberts, Kelly Sadler, Tina Stavros, Roy Tamura, Keith Wood, Ping Xu, Kenneth Young. Past Staff Members: Persida Alies, Franz Badias, Aaron Baker, Monica Bassi, Craig Beam, London Bounmananh, Susan Bream, Doug Freeman, Jessica Gough, Jinin Ginem, Moriah Granger, Mary Holloway Michelle Kieffer, Page Lane, Lavanya Nallamshetty, Yazandra Parrimon, Kate Paulus, Joy Ramiro, AQesha Luvon Ritzie, Archana Sharma, Audrey Shor, Xiaohong Song, Amanda Terry, Jeanne Weinberger, Margaret Wootten.

Previous Coordinating Center (George Washington University): John M. Lachin, Mary Foulkes, Pamela Harding, Heidi Krause-Steinrauf, Susan McDonough, Paula F. McGee, Kimberly Owens Hess, Donna Phoebus, Scott Quinlan, Erica Raiden.

NIDDK Staff: Judith Fradkin, Ellen Leschek, Lisa Spain. Past Member: Peter Savage.

Data Safety and Monitoring Board: Gerald Beck (Cleveland Clinic), Emily Blumberg (University of Pennsylvania), Chair, David Brillon (Cornell University), Rose Gubitosi-Klug (Case Western Reserve), Lori Laffel (Joslin Diabetes Center), Robert Veatch (Georgetown University), Dennis Wallace (Research Triangle Institute). Past Members: Jonathan Braun (University of California Los Angeles), Ake Lernmark (Lund University), Bernard Lo (University of California San Francisco), Herman Mitchell (Rho Inc.), Ali Naji (University of Pennsylvania), Jorn Nerup (University of Copenhagen), Trevor Orchard (University of Pittsburgh), Michael Steffes (University of Minnesota), Anastasios Tsiatis (North Carolina State University), Bernard Zinman (University of Toronto).

Infectious Disease Safety Committee: Brett Loechelt (Children's National Medical Center) (Medical Monitor), Lindsey Baden (Harvard University), Michael Green (University of Pittsburgh), Adriana Weinberg (University of Colorado).

Laboratory Directors: Santica Marcovina (University of Washington), Jerry P. Palmer, Adriana Weinberg, Liping Yu (University of Colorado Barbara Davis Center for Childhood Diabetes), Sunanda Babu (University of Colorado Barbara Davis Center for Childhood Diabetes) William Winter (University of Florida). Past Member: George S. Eisenbarth (late).

Protocol Chair Committee: Polly Bingley, Raphael Clynes, Linda DiMeglio, George Eisenbarth, Carla Greenbaum, Brian Hays, Jeffrey Krischer, Ellen Leschek, Jennifer Marks, Della Matheson, Lisa Rafkin, Henry Rodriguez, Jay Skyler, Jay Sosenko, Lisa Spain, Darrell Wilson.

Clinical Center Staff Involved in this Protocol:

Baylor College of Medicine: Maria Redondo, David Gomez, Andrene McDonald, Sandra Pena, Massimo Pietropaolo, Kathy Shippy.

Benaroya Research Institute, Seattle: Carla Greenbaum, Emily Batts, Tyler Brown, Jane Buckner, Angela Dove, Marissa Hammond, Deborah Hefty, Jani Klein, Kristen Kuhns, McKenzie Letlau, Sandra Lord, Marli McCulloch-Olson, Lisa Miller, Gerald Nepom, Jared Odegard, Mary Ramey, Elaine Sachter, Marissa St. Marie, Kimberly Stickney, Dana VanBuecken, Ben Vellek, Christine Webber. Past Members: Laurie Allen, Jenna Bollyk, Nicole Hilderman, Hebatullah Ismail, Steve Lamola, Srinath Sanda, Heather Vendettuoli, David Tridgell.

Children's Hospital Los Angeles: Roshanak Monzavi, Meredith Bock, Lynda Fisher, Mary Halvorson, Debra Jeandron, Mimi Kim, Jamie Wood. Past Members: Mitchell Geffner, Francine Kaufman, Robertson Parkman, Christine Salazar.

Columbia University, New York: Robin Goland, Raphael Clynes, Steve Cook, Matthew Freeby, Mary Pat Gallagher, Rachelle Gandica, Ellen Greenberg, Amy Kurland, Sarah Pollak, Amy Wolk. Past Members: Mary Chan, Linda Koplimae, Elizabeth Levine, Kelly Smith, Jeniece Trast.

Indiana University, Indianapolis: Linda DiMeglio, Janice Blum, Carmella Evans-Molina, Robin Hufferd, Bonnie Jagielo, Christy Kruse, Vanessa Patrick, Mark Rigby, Maria Spall, Kim Swinney, Jennifer Terrell. Past Members: Lyla Christner, Lee Ann Ford, Sheryl Lynch, Martha Menendez, Patricia Merrill, Mark Pescovitz (late), Henry Rodriguez.

Joslin Diabetes Center, Boston: Cielo Alleyn, David Baidal, Steve Fay, Jason Gaglia, Brittany Resnick, Sarah Szubowicz, Gordon Weir. Past Members: Ronald Benjamin, Debbie Conboy, Andrea de Manbey, Richard Jackson, Heyam Jalahej, Tihmar Orban, Alyne Ricker, Joseph Wolfsdorf, Hui H. Zhang.

Stanford University: Darrell Wilson, Tandy Aye, Bonita Baker, Karen Barahona, Bruce Buckingham, Kerry Esrey, Trudy Esrey, Garry Fathman, Radhika Snyder. Past Members: Beenu Aneja, Maya Chatav, Oralia Espinoza, Eliana Frank, Jenny Liu, Jennifer Perry, Rebecca Pyle, Alison Rigby, Kristin Riley, Adriana Soto.

University of California San Francisco: Stephen Gitelman, Saleh Adi, Mark Anderson, Ashley Berhel, Kathy Breen, Kathleen Fraser, Andrea Gerard-Gonzalez, Paula Jossan, Robert Lustig, Sara Moassesfar, Amy Mugg, David Ng, Priya Prahalod, Martha Rangel-Lugo, Srinath Sanda, Joshua Tarkoff, Christine Torok, Rebecca Wesch. Past Members: Ivy Aslan, Jeanne Buchanan, Jennifer Cordier, Celia Hamilton, Louise Hawkins, Thu Ho, Anjali Jain, Karen Ko, Theresa Lee, Shelly Phelps, Stephen Rosenthal, Taninee Sahakitrungruang, Lorraine Stehl, Lisa Taylor, Marcia Wertz, Jenise Wong.

University of Chicago: Louis Philipson, Rosemary Briars, Nancy Devine, Elizabeth Littlejohn. Past Member: Tiffany Grant.

University of Colorado Barbara Davis Center for Childhood Diabetes, Denver: Peter Gottlieb, Georgeanna Klingensmith, Andrea Steck, Aimon Alkanani, Kimberly Bautista, Ruth Bedoy, Aaron Blau, Betsy Burke, Laraine Cory, My Linh Dang, Lisa Fitzgerald-Miller, Alex Fouts, Vicky Gage, Satish Garg, Patricia Gesauldo, Raymond Gutin, Cory Hayes, Michelle Hoffman, Kaitlin Ketchum, Nyla Logsden-Sackett, David Maahs, Laurel Messer, Lisa Meyers, Aaron Michels, Stesha Peacock, Marian Rewers, Perla Rodriguez, Flor Sepulbeda, Rachel Sippl, Andrea Steck, Iman Taki, Bao-Khan Tran, Tuan Tran, R. Paul Wadwa, Philip Zeitler. Past Members: Jennifer Barker, Sandra Barry, Laurie Birks, Leah Bomsburger, Terra Bookert, Leah Briggs, Patricia Burdick, Rosio Cabrera, Peter Chase, Erin Cobry, Amy Conley, Gabrielle Cook, Joseph Daniels, Dominic Di Domenico, Jennifer Eckert, Angelica Ehler, George Eisenbarth (late), Pamela Fain, Rosanna Fiallo-Scharer, Nicole Frank, Hannah Goettle, Michelle Haarhues, Sherrie Harris, Lauren Horton, John Hutton (late), Joy Jeffrrey, Rachael Jenison, Kelly Jones, Whitney Kastelic, Maria Amelia King, Debbie Lehr, Jenna Lungaro, Kendra Mason, Heather Maurer, Luy Nguyen, Allison Proto, Jaime Realsen, Kristina Schmitt, Mara Schwartz, San Skovgaard, Jennifer Smith, Brandon Vanderwel, Mary Voelmle, Rebecca Wagner, Amy Wallace, Philip Walravens, Laurie Weiner, Becky Westerhoff, Emily Westfall, Katina Widmer, Hali Wright.

University of Florida, Gainesville: Desmond Schatz, Annie Abraham, Mark Atkinson, Miriam Cintron, Michael Clare- Salzler, Jessica Ferguson, Michael Haller, Jennifer Hosford, Diane Mancini, Hank Rohrs, Janet Silverstein, Jamie Thomas, William Winter. Past Members: Gloria Cole, Roberta Cook, Ryan Coy, Elena Hicks, Nancy Lewis.

University of Miami: Jennifer Marks, Alberto Pugliese, Carlos Blaschke, Della Matheson, Alberto Pugliese, Natalia Sanders-Branca, Jay Sosenko. Past Members: Luz Arazo Ray Arce, Mario Cisneros, Samir Sabbag.

University of Minnesota, Minneapolis: Antoinette Moran, Carrie Gibson, Brian Fife, Bernhard Hering, Christine Kwong, Janice Leschyshyn, Brandon Nathan, Beth Pappenfus, Anne Street. Past Members: Mary Ann Boes, Sarah Peterson Eck, Lois Finney, Theresa Albright Fischer, Andrea Martin, Chenai Jacqueline Muzamhindo, Missy Rhodes, Jennifer Smith, John Wagner, Bryan Wood.

University of Pittsburgh: Dorothy Becker, Kelli Delallo, Ana Diaz, Barbara Elnyczky, Ingrid Libman, Beata Pasek, Karen Riley, Massimo Trucco. Past Members: Brian Copemen, Diane Gwynn, Frederico Toledo.

University of South Florida: Henry Rodriguez, Sureka Bollepalli, Frank Diamond, Emily Eyth, Danielle Henson, Anne Lenz, Dorothy Shulman.

University of Texas Southwestern, Dallas: Phillip Raskin, Soumya Adhikari, Brian Dickson, Erin Dunnigan, Ildiko Lingvay, Lourdes Pruneda, Maria Ramos-Roman, Chanhaeng Rhee, John Richard, Mark Siegelman, Daytheon Sturges, Kathryn Sumpter, Perrin White. Past Members: Marilyn Alford, Jamie Arthur, M. Larissa Aviles-Santa, Erica Cordova, Renee Davis, Stefani Fernandez, Steve Fordan, Tauri Hardin, Aris Jacobs,

Polina Kaloyanova, Ivanna Lukacova Zib, Sasan Mirfakhraee, Alok Mohan, Hiroshi Noto, Oralenda Smith, Nenita Torres.

University of Toronto: Diane Wherrett, Diana Balmer, Lesley Eisel, Roze Kovalakovska, Mala Mehan, Farah Sultan. Past Members: Brenda Ahenkorah, Jose Cevallos, Natasha Razack, Mary Jo Ricci, Angela Rhode, Mithula Srikandarajah, Rachel Steger.

Vanderbilt University, Nashville: William E. Russell, Margo Black, Faith Brendle, Anne Brown, Daniel Moore, Eric Pittel, Alyssa Robertson, April Shannon, James W. Thomas.

Yale University, New Haven: Kevan Herold, Laurie Feldman, Robert Sherwin, William Tamborlane, Stuart Weinzimer.

International Clinical Center Staff involved in this Protocol:

Hospital District of Southwest Finland: Jorma Toppari, Tiina Kallio, Maarit Kärkkäinen, Elina Mäntymäki, Tiina Niininen, Birgitta Nurmi, Petro Rajala, Minna Romo, Sointu Suomenrinne. Past Members: Kirsti Näntö- Salonen, Olli Simell, Tuula Simell.

San Raffaele Hospital, Milan (Italy): Emanuele Bosi, Manuela Battaglia, Eleonora Bianconi, Riccardo Bonfanti, Pauline Grogan, Andrea Laurenzi, Sabina Martinenghi, Franco Meschi, Matteo Pastore. Past Members: Luca Falqui, Maria Teresa Muscato, Matteo Viscardi.

University of Bristol (United Kingdom): Polly Bingley, Harriet Castleden, Nicola Farthing, Sam Loud, Pilar Munoz, Beth Thorne. Past Members: Rebecca Elliot-Jones, Claire Matthews, Ann Morgan, Jennifer McGhee, Joanna Pollitt, Carole Wheaton.

University of Helsinki: Mikael Knip, Heli Siljander, Heli Suomalainen.

Walter and Eliza Hall Institute of Medical Research (Australia): Peter Colman, Felicity Healy, Shelley Mesfin, Leanne Redl, John Wentworth, Jinny Willis. Past Members: Maree Farley, Leonard Harrison, Christine Perry, Fiona Williams.
